# Growth of young HIV-infected and HIV-exposed children in western Kenya: A retrospective chart review

**DOI:** 10.1371/journal.pone.0224295

**Published:** 2019-12-04

**Authors:** Megan S. McHenry, Edith Apondi, Samuel O. Ayaya, Ziyi Yang, Wenfang Li, Wanzhu Tu, Guanying Bi, Edwin Sang, Rachel C. Vreeman

**Affiliations:** 1 Department of Pediatrics, Indiana University School of Medicine, Indianapolis, Indiana, United States of America; 2 Academic Model Providing Access to Healthcare (AMPATH), Eldoret, Kenya; 3 Department of Child Health and Paediatrics, College of Health Sciences, School of Medicine, Moi University, Eldoret, Kenya; 4 Department of Biostatistics, Indiana University School of Medicine, Indianapolis, Indiana, United States of America; 5 Department of Biostatistics, Indiana University Fairbanks School of Public Health, Indianapolis, Indiana, United States of America; Sefako Makgatho Health Sciences University, SOUTH AFRICA

## Abstract

**Introduction:**

The objective of this study was to determine the growth patterns, rates of malnutrition, and factors associated with malnutrition in children born to HIV-infected mothers in western Kenya using data from an electronic medical record system.

**Methods:**

This study was a retrospective chart review of HIV-infected (HIV+) and–exposed (HEU) children (<5 years) using data collected prospectively in the course of routine clinical care and stored in the electronic medical record system in western Kenya between January 2011 and August 2016. Demographics and anthropometrics were described, with Chi-square testing to compare proportions. Multiple variable logistic regression analysis was used to identify correlates of children being stunted, underweight, and wasted. We also examined growth curves, using a resampling method to compare the areas under the fitted growth curves to compare males/females and HIV+/HEU.

**Results:**

Data from 15,428 children were analyzed. The children were 51.6% (n = 7,955) female, 5.2% (n = 809) orphans, 83.3% (n = 12,851) were HEU, and 16.7% (n = 2,577) were HIV+. For HIV+ children assessed at 24 months, 50.9% (n = 217) were stunted, 26.5% (n = 145) were underweight, and 13.6% (n = 58) were wasted, while 45.0% (n = 577) of HEU children were stunted, 14.8% (n = 255) were underweight, and 5.1% (n = 65) were wasted. When comparing mean z-scores, HIV+ children tended to have larger and earlier dips in z-scores compared to HIV-exposed children, with significant differences found between the two groups (p<0.001). Factors associated with an increased risk of malnutrition included being male, HIV+, and attending an urban clinic. Maternal antiretroviral treatment during pregnancy and mixed feeding at 3 months of age decreased the risk of malnutrition.

**Conclusions:**

HIV+ and HEU children differ in their anthropometrics, with HIV+ children having overall lower z-scores. Continued efforts to develop and implement sustainable and effective interventions for malnutrition are needed for children born to HIV+ mothers.

## Introduction

Malnutrition contributes to nearly 50% of the deaths in children under 5 years of age worldwide, which translates into 3 million deaths per year [1]. Children with malnutrition are caught in a vicious cycle–their poor nutritional state puts them at increased risk of infection, then acute or chronic illness further depletes their valuable nutrients and their risk of infection increases even more [2]. Moreover, stunted growth is associated with impaired cognitive ability and reduced school and work performance [2–5]. In many African countries, like Kenya, over a third of children have moderate-to-severe stunting [6].

Compounding the issue of malnutrition in these settings is the prevalence of HIV. Children born to HIV-infected (HIV+) mothers are more likely to be malnourished [7–9]. Poor growth in HIV+ children contributes to immune dysfunction and is associated with disease progression and decreased survival [10, 11]. Even those who are HIV-uninfected but were born to mothers with HIV have increased risks of mortality and morbidity compared to their HIV-unexposed counterparts. In a study performed in Zimbabwe, nearly 80% of children infected with HIV+ *in utero* and 35% of HEU children were stunted at 24 months [8]. These increased risks are thought to be related to *in utero* and early life exposures, increased risk of prematurity, and reduced care due to parental illness or death [12–15].

Increased implementation of strong electronic medical records (EMR) systems in resource-limited settings provides the opportunity to obtain real-world clinical data on a large number of children more easily than in decades past [16, 17]. While our current knowledge of the nutritional status of HIV+ and HIV–exposed but uninfected (HEU) children is informed by prospective studies, these studies are resource-intense, challenging to replicate, and only represent a subset of the population [8, 9, 14, 18]. Utilizing EMR systems to obtain real-time data for specific variables, such as rates of malnutrition, enable healthcare systems to identify issues most negatively impacting their patients.

In this study, we used a large retrospective data set from a healthcare system in western Kenya to examine the growth data for young children born to HIV+ mothers to determine the growth patterns, rates of malnutrition, and factors associated with malnutrition in children born to HIV+ mothers.

## Methods

### Study design and setting

This is a retrospective study using data collected prospectively in the course of routine clinical care and stored in the electronic medical record (EMR) system. Data were pulled for all children who were <5 years of age between January 2011- August 2016 and enrolled in a large HIV clinical care system in Kenya, the Academic Model Providing Access to Healthcare (AMPATH). Born from a 20-year partnership between Indiana University School of Medicine (IUSM), Moi University School of Medicine (MUSM), and the Moi Teaching and Referral Hospital in Eldoret, Kenya, the AMPATH HIV care program has enrolled over 160,000 patients and currently provides care for approximately 15,000 HIV+ and HEU children in 65 clinics in western Kenya [19]. During this period, AMPATH clinical data were captured on standardized paper encounter forms and then entered into the AMPATH Medical Records System (AMRS), a resource for both patient care and research evaluations. AMRS was sub-Saharan Africa’s first comprehensive EMR for HIV care, pioneering the effective use of EMRs in such settings [20, 21]. Outcomes of HIV+ children in this large cohort have previously been reported for retention in care, therapy, and HIV transmission rates [22–24].

This study was approved by the IUSM Institutional Review Board and the United States’ Office of Human Research Protections-approved MUSM Institutional Research and Ethics Committee. The requirement for informed consent was waived by both ethical governing bodies. The data for this study were handled and stored within Health Insurance Portability and Accountability Act of 1996-compliant secured servers.

### Study participants

Participants were eligible if they were seen in any of the AMPATH clinics between January 2011 and August 2016, were <5 years of age when enrolling in care during that time period, were born to HIV+ mothers, and had at least one anthropometric measurement recorded. In this setting, HIV-exposed children receive confirmatory HIV testing at 18 months of life. Because this dataset encompassed data from a specific range of time, there are individuals who were monitored by AMPATH but had not yet reached 18 months of age prior to completion of data collection. This group was termed “HIV-indeterminate” for this study. Also included in this group were the individuals who were lost-to-follow-up prior to 18 months of age. HIV-indeterminate children (n = 1,576) were removed from the dataset. All children who were exposed to HIV but had negative confirmatory testing during the data collection period are referred to as being HEU. HEU children were eligible to receive free follow-up care with well-child visits in the AMPATH system until 5 years of age. No HIV-unexposed children are included in this cohort.

Per AMPATH and Kenya’s Ministry of Health protocols, during the period of data collection, pregnant mothers with HIV were expected to be on an antiretroviral treatment (ART) regimen of tenofovir, lamivudine, and efavirenz as the first-line combination (Option B+) which would continue for life. Exclusive breastfeeding was recommended for all HIV-exposed children for the first 6 months of life and to continue breastfeeding with appropriate complementary feeding introduced thereafter [25]. Nevirapine prophylaxis was given for 6 weeks to all infants attending the AMPATH clinics and co-trimoxazole was given from 6 weeks of age until the time of confirmatory testing at 18 months of age [25]. If a child was found to be HIV+ at any time, they would be switched to either a regimen of zidovudine/lamivudine/nevirapine or abacavir/lamivudine/nevirapine as first-line treatment. In late 2013, the first-line regimen for children was changed to abacavir/lamivudine/lopinavir/ritonavir, in accordance with changes in the World Health Organization (WHO) guidelines [26]. Per AMPATH standard operating procedures, from 2010- December 2015 all children included in this study were recommended to attend monthly follow-up visits until 5 years of age [27]. In 2016, this was changed to be in line with the national policy of monthly visits until 18 month and then every 6 months until 5 years of age [25].

### Data collection

The variables included within this study were collected from clinic visit forms completed by a clinical officer (a mid-level provider), medical officer, or pediatrician and entered into the AMRS system. These variables included age at enrollment, sex, clinic location/type, person accompanying child, orphan status, visit height/weights, caregiver-reported feeding method, clinician-reported maternal or child ART, HIV+ sibling, mean CD4 for HIV+ children, and final HIV testing result. There were also places where a clinician could indicate whether they believed a child was considered developmentally delayed or failure to thrive, although no standard operating procedures or guidelines were available outlining the definitions of those terms. Patient identifiers, including name, address, and contact information, were removed during data extraction. All analyzed data were handled and transferred using password protected, encrypted methodologies compliant with the United States’ Health Insurance Portability and Accountability Act standards.

For this study, z-scores and standardized WHO definitions were used for presentation and analysis of anthropometric data, with z-scores calculated using the modeling defined by the WHO [28]. Z-scores are expressed anthropometric values as several standard deviations below or above the reference mean or median values, that are helpful for grouping growth data by age and sex [29]. To characterize malnutrition, the following categories were evaluated: stunting, underweight, and wasting. In these analyses, “stunting” refers to moderate-to-severe stunting, (height-for-age (HFA) z-scores of ≤-2). “Underweight” refers to moderate-to-severe underweight status (weight-for-age (WFA) z-scores of ≤-2). “Wasting” refers to moderate-to-severe wasting (weight-for-height (WFH) z-scores of ≤-2). The WHO defines “moderate-severe malnutrition” based on these three variables.

### Statistical analysis

To minimize the influences of recording errors and data irregularity, we conducted a due-diligence examination of the height and weight growth data: We restricted the weight change to no more than +/-3 kg per month, the height change to less than 10cm per month. We excluded height measures that were shorter than previously recorded heights, and z-scores (WFA, HFA, WFH) that changed more than +/-2 units per month. These thresholds were determined upon review of World Health Organization growth charts, review of the current dataset, and by using clinical judgement.

Participant characteristics at study entry were summarized in a tabular form. Frequencies and percentages were calculated for categorical variables. Any visits occurring at a certain age point included a +/- 1 month window. For example, children who came to clinic between the ages of 11 and 13 months were marked as having a 12 month visit. In the event that multiple visits occurred within that window, only the first visit’s data was included. Mean and standard deviation were calculated for continuous variables. Descriptive statistics for participant characteristics were evaluated for the full sample and for the subsamples defined by the HIV status. Comparisons between HIV+ and HEU children of characteristics presented as proportions were analyzed using Chi-squared test, while comparisons of means between the two groups were analyzed using independent t-tests.

We also compared the proportions of stunting, underweight, and wasting among HIV+ and HEU children, using chi-square tests. We then performed a visit-level analysis, assessing WFA, HFA, and WFH at multiple points of observations. A random effects logistic regression model was used to accommodate within-subject correlations over time to assess the effects of factors that were associated with the risks of stunting, underweight, and wasting. Two authors (M.S.M. and R.C.V) reviewed all available variables and selected those which may be potential confounders using clinical judgement and knowledge of the literature. Univariate analysis was then performed with each of these variables, and those found to reach statistical significance (α set at 0.05) were included in the multiple variable logistical regression models. Estimated adjusted odds ratios and related confidence intervals were reported. Finally, we comparatively examined the mean HFA, WFA, and WFH curves among the two HIV subgroups, over the entire age range. Comparisons of the curves were made using a resampling-based test [30].

All analyses were implemented in SAS 9.4 (SAS Institute, Cary NC) and R 3.4.0. P values less than 0.05 were considered statistically significant.

## Results

### Participant characteristics

**Fig 1:**
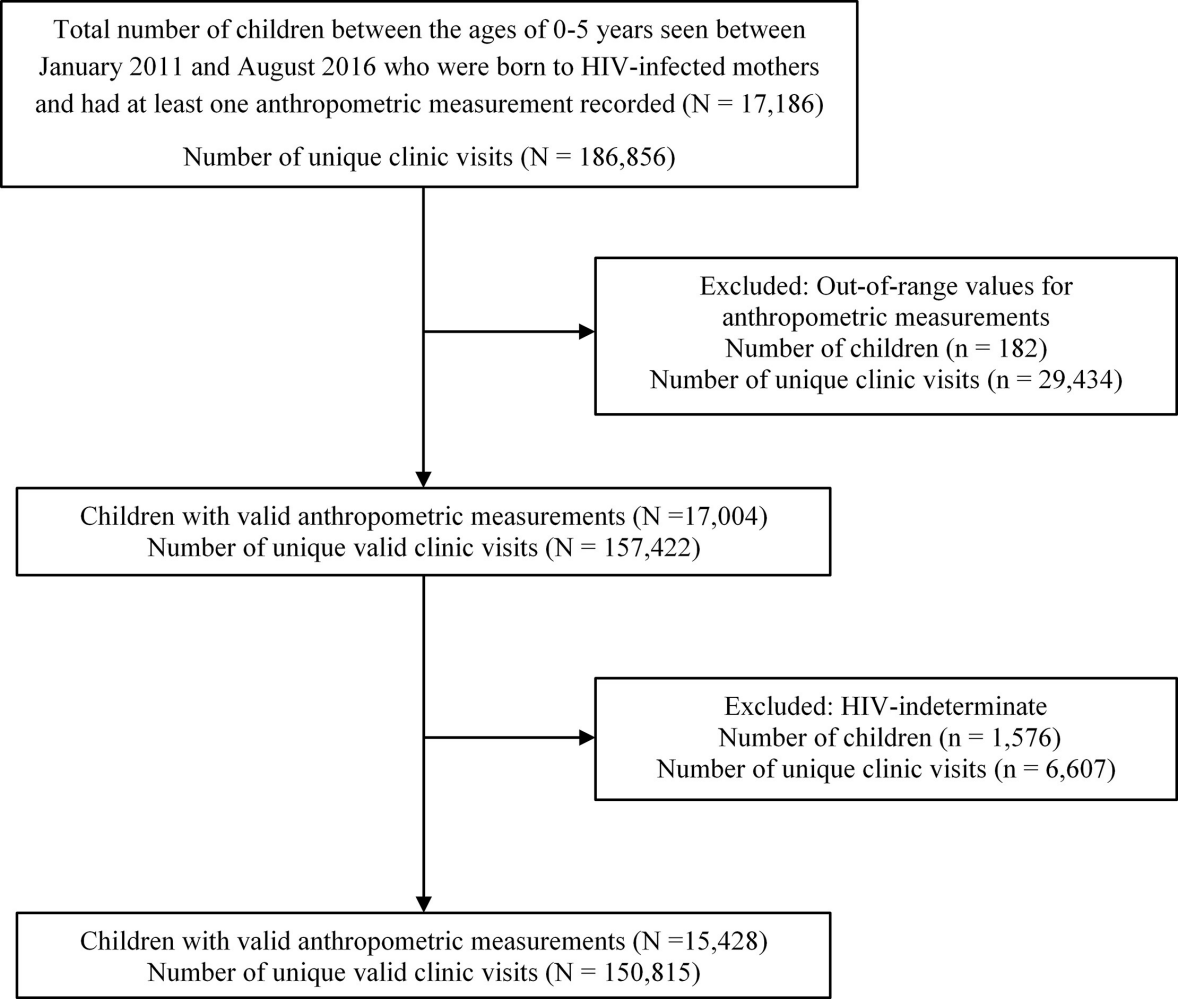
Flow diagram of study population selection.

During the time period of interest, 15,428 children met the inclusion criteria, with 150,815 clinic visits included among them (Fig 1). Fifty-two percent (n = 7,955) of children were female, 16.7% (n = 2,577) were HIV+, and 83.3% (n = 12,851) were HEU. The mean age of enrollment was 2.1 ± 1.5 years for HIV+ children and 0.2 ± 0.3 years for HEU children. Nearly 15% (n = 372) of HIV+ children had at least one deceased parent compared to 3.5% (n = 437) of HEU children. Only 1% (n = 151) of all children were identified as failure to thrive by the clinician and 1.2% (n = 189) of all children were indicated to have a developmental disability by the clinician assessing them (Table 1). Baseline characteristics between HIV+ and HEU were significantly different from one another (most with p <0.001), with the exception of sex and specific clinic locations. For those HIV+ children with CD4 counts (n = 700), the mean and standard deviation was 798.5 and 471.5, respectively.

**Table 1:** Study participant characteristics.

	Total: N = 15428 n (%)	HIV- infected: n = 2577 n (%)	HIV-exposed but uninfected: n = 12851 n (%)	P-value
**Age at enrollment- mean (SD)**	0.5 (1.0)	2.1 (1.5)	0.2 (0.3)	**< 0.001**
**Sex- (% female)**	7955 (51.6)	1320 (51.2)	6635 (51.6)	0.705
**Clinic Location**				
Referral hospital	2525 (16.7)	382 (15.2)	2143 (17.0)	
County/Sub-County Level	6938 (45.8)	1141 (45.5)	5797 (45.9)	
Rural Health Clinic	5672 (37.5)	985 (39.3)	4687 (37.1)	**0.039**
**Orphaned**				
Only mother deceased	231 (1.5)	146 (5.7)	85 (0.7)	
Only father deceased	231 (1.5)	179 (7.0)	319 (2.5)	
Both deceased	80 (0.5)	47 (1.8)	33 (0.3)	
Both alive	14619 (94.8)	2205 (85.6)	12414 (96.6)	**< 0.001**
**Maternal ART given during pregnancy**	6670 (43.2)	226 (8.8)	6444 (50.1)	**< 0.001**
**Child received ART prophylaxis or treatment**	7424 (54.4)	237 (10.8)	7187 (62.7)	**< 0.001**
**Failure to Thrive by Clinician Assessment**	151 (1.0)	107 (4.2)	44 (0.3)	**< 0.001**
**Developmental delay or disability by Clinician Assessment**	189 (1.2)	129 (5.0)	60 (0.5)	**< 0.001**
**Feeding method at 3 months**^**a**^				
Exclusive breastfeeding	6373 (83.4)	304 (79.0)	6069 (83.6)	**0.017**
Expressed breast milk	26 (0.3)	3 (0.8)	23 (0.3)	0.129
Formula feeding	380 (5.0)	51 (13.3)	329 (4.5)	**< 0.001**
Feeding solids	1212 (15.9)	79 (20.5)	1133 (15.6)	**0.010**
Feeding water or other liquids	632 (8.3)	87 (22.6)	545 (7.5)	**< 0.001**
**Sibling with HIV**	940 (12.1)	216 (16.7)	724 (11.2)	**< 0.001**
**Mean CD4 –mean (SD**)	--	798.5 (471.5) n = 700	--	--

^a^- Denominator equals those with feeding method listed at 3 months. Total sample, n = 7644; HIV-infected, n = 385; HIV-exposed but uninfected, n = 7259

### Rates of malnutrition

When compared to HEU children, the HIV+ children had higher rates of stunting, underweight status, and wasting at multiple time points. This difference was most significant for stunting at the 12 months visit (p<0.001); 24 month visit (p = 0.035); 36 month visit (p<0.001); and 48 month visit (p = 0.002). This difference was also statistically significant when comparing underweight status at the 12 month visit (p<0.001); 24 month visit (p<0.001); and 36 month visit (p = 0.004) and for wasting at 12 months (p<0.001) and 24 months (p<0.001) (Table 2). The numbers of HEU children coming in for visits decreased over time, with the largest decreases in attendance after the 12 and 24 month visits.

**Table 2:** The percentage of malnourished HIV-infected and HIV-exposed children, by age of child.

	HIV-infected n/total† (%)	HIV-exposed n (%)	p-value
Stunted (HFA)			
12 month visit	181/368 (49.2)	1873/5363 (34.9)	**<0.001**
24 month visit	217/426 (50.9)	577/1281 (45.0)	**0.035**
36 month visit	232/483 (48.0)	111/362 (30.7)	**<0.001**
48 month visit	204/526 (38.8)	34/138 (24.6)	**0.002**
60 month visit	118/336 (35.1)	3/11 (27.3)	0.754
Underweight (WFA)			
12 month visit	157/503 (31.2)	946/7214 (13.1)	**<0.001**
24 month visit	145/548 (26.5)	255/1718 (14.8)	**<0.001**
36 month visit	131/601 (21.8)	68/459 (14.8)	**0.004**
48 month visit	118/624 (18.9)	26/163 (16.0)	0.384
60 month visit	87/396 (22.0)	2/12 (16.7)	>0.999
Wasted (WFH)			
12 month visit	78/372 (21.0)	431/5383 (8.1)	**<0.001**
24 month visit	58/426 (13.6)	65/1277 (5.1)	**<0.001**
36 month visit	36/481 (7.5)	17/357 (4.8)	0.109
48 month visit	38/515 (7.4)	12/138 (8.7)	0.605
60 month visit	31/326 (9.5)	1/11 (9.1)	>0.999

† represents the total number of children who came to clinic +/- 1 month the specific age visit. For example, children coming to clinic between the ages of 11 and 13 months were included in the data reported for the 12 month visit. Bolded numbers indicate those below α 0.05, indicating statistical significance.

### Trends of growth over time

**Fig 2:**
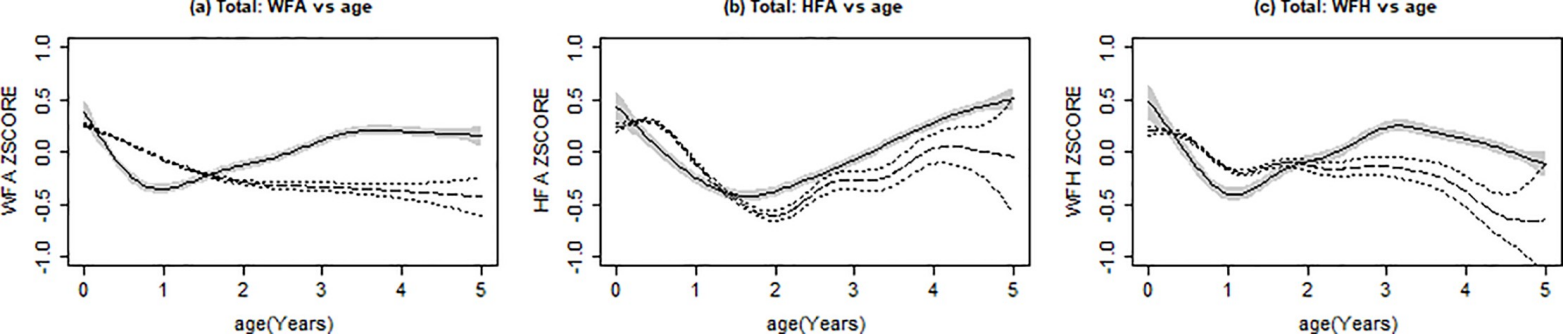
Growth trends over time, by HIV status. Solid line with shading indicates mean z-score for HIV-infected children. Dotted lines indicates mean z-score HIV-exposed but uninfected children. Shaded areas and dotted lines indicate 95% confidence interval of the estimated mean. (A) Weight-for-age (WFA) z-scores over age, in years; (B) Height-for-age (HFA) z-scores over age, in years; (C) Weight-for-height (WFH) z-scores over age, in years.

**Fig 3:**
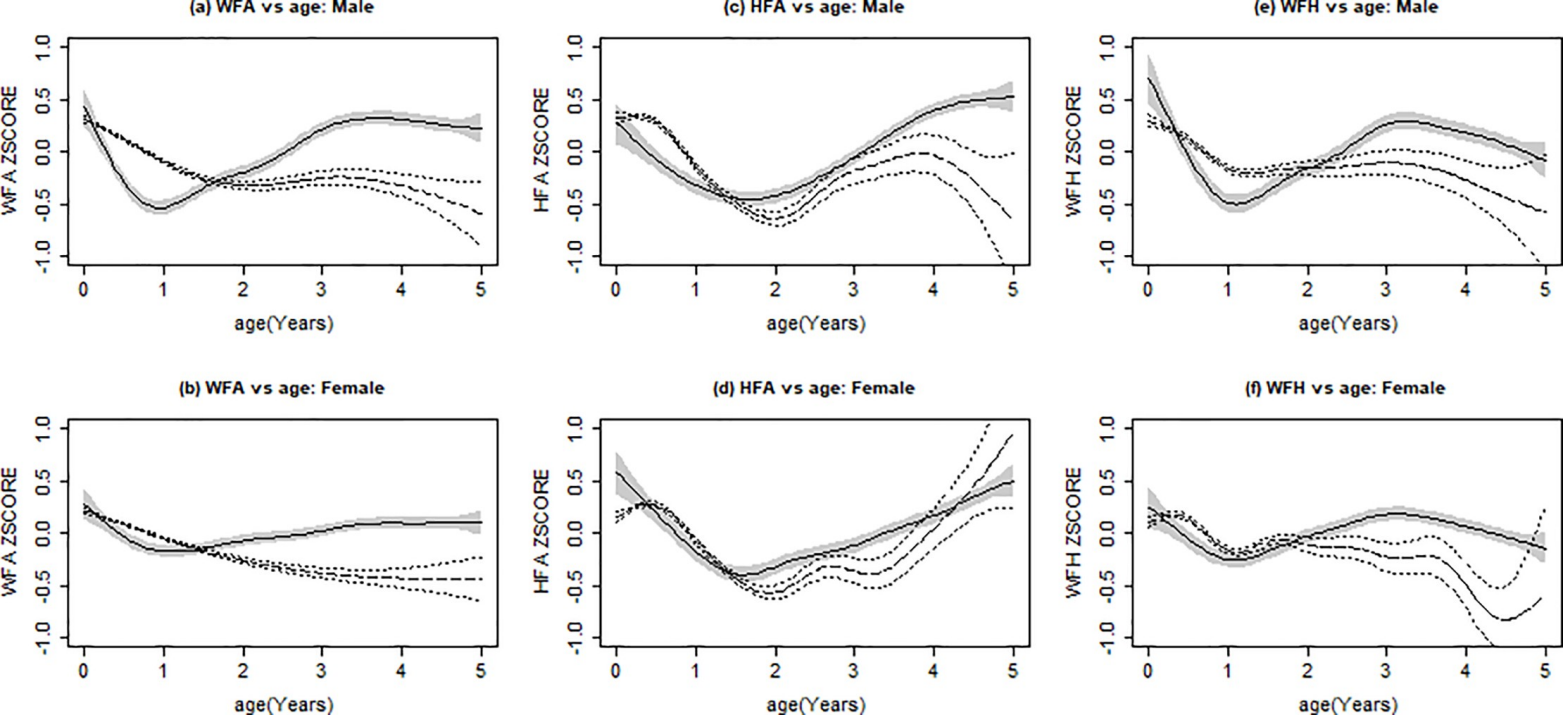
Growth trends over time, by HIV status and sex. Solid line with shading indicates mean Z-score for HIV-infected children. Dotted lines indicates mean Z-score HIV-exposed but uninfected children. Shaded areas and dotted lines indicate 95% confidence interval of the estimated mean. (A) Weight-for-age (WFA) z-scores over age, males only; (B) WFA z-scores over age, females only; (C) Height-for-age (HFA) z-scores over age, males only; (D) HFA z-scores over age, females only; (E) Weight-for-height (WFH) z-scores over age, males only; (F) WFH z-scores over age, females only.

Growth trends over time are illustrated by HIV status (Fig 2) and further subdivided by sex (Fig 3).

#### Underweight

HIV+ children had a sharp decrease in WFA z-scores during the first year of their life (Fig 2A), and this was most prominent among the HIV+ males with a low estimated mean of -0.534 at 11 months of age (Fig 3A). This decrease in WFA z-scores gradually improved over time, and by 20 months, HIV+ children, both male and female, had a higher mean WFA z-score compared to HEU children. Overall, the growth curves of the HIV+ and HEU WFA z-scores were significantly different from each other, with HEU generally having lower z-scores (p <0.001).

#### Stunting

The decline in the HFA z-scores started after birth for HIV+ children and around 6 months of age for HEU, reaching a low of -0.380 at 18 months for HIV+ children and -0.417 at 24 months for HEU children. Catch-up growth occurred for both HIV+ and HEU children, although the HEU caught up at a slower rate (Fig 2B). When comparing male and female HFA z-scores over time, their trends were similar (Fig 3C and 3D). Overall, there was a significant difference between HIV+ and HEU HFA z-score growth curves (p <0.001).

#### Wasting

WFH z-scores followed a similar pattern to that of WFA z-scores. HIV+ children had a sharp decrease in WFH z-scores early in life, reaching an estimated mean low of -0.403 at 12.7 months of age (Fig 2C); however, the sharp decline seen among HIV+ males, a mean low of -0.497 at 13.3 months, likely influenced the severity of this drop (Fig 3E). This decrease gradually improved, and by 24 months, HIV+ children had a higher mean WFA z-scores compared to HEU. Overall, there was a significant difference between the HIV+ and HEU WFA growth curves, with HEU having lower z-scores (p <0.001).

### Factors associated with malnutrition

Children living in rural settings were less likely to be stunted (OR:0.45; 95%CI:0.41 to 0.50) compared to those living in urban settings. The odds of being stunted when attending a rural health clinic or county/sub-county hospital were much higher compared to those for children attending the referral hospital, (county/sub-county: (OR:3.88;95%CI:3.53 to 4.27); rural health clinic: (OR:5.06; 95%CI:4.42 to 5.80)).

Children accompanied to clinic by grandparents were more likely to be underweight (OR:1.49; 95%CI:1.25 to 1.77) and wasted (OR:1.45; 95%CI:1.11 to 1.79). For every year of age older a child was enrolled in clinic, there was a higher odds of being stunted (OR:1.17; 95%CI:1.11 to 1.23) and underweight (OR:1.25; 95%CI:1.18 to 1.32). Females and those with ART exposure *in-utero* were less likely to be stunted, underweight, or wasted, while children who were HIV+ were more likely to be malnourished. Children for whom families reported mixed feedings at 3 months of age were less likely to be malnourished (Table 3).

**Table 3:** Factors associated with stunted, underweight, and wasted status.

	Adjusted Odds Ratio (95% CI)		
Variable	Stunted (HFA Z-score ≤ -2)	Underweight (WFA Z-score ≤ -2)	Wasted (WFH Z-score ≤ -2)
**Age at enrollment**	**1.17 (1.11, 1.23)**	**1.25 (1.18, 1.32)**	**0.88 (0.83, 0.94)**
**Setting**			
Urban	Reference	Reference	Reference
Rural	**0.45 (0.41, 0.50)**	1.00 (0.89, 1.13)	**1.84 (1.59, 2.10)**
**Clinic location**			
Referral hospital	Reference	Reference	Reference
County/sub-county hospital	**3.88 (3.53, 4.27)**	0.95 (0.85, 1.07)	**0.43 (0.38, 0.48)**
Rural health clinic	**5.06 (4.42, 5.80)**	1.03 (0.88, 1.21)	**0.41 (0.34, 0.48)**
**Female**	**0.70 (0.65, 0.75)**	**0.71 (0.65, 0.76)**	**0.82 (0.76, 0.89)**
**Orphan status**			
Both alive	Reference	Reference	Reference
Only father deceased	1.09 (0.98, 1.21)	**1.13 (1.004, 1.27)**	1.11 (0.97, 1.26)
Only mother deceased	**1.32 (1.11, 1.56)**	1.10 (0.92, 1.32)	**0.79 (0.60, 0.98)**
Both deceased	0.91 (0.75, 1.12)	**0.67 (0.54, 0.83)**	0.82 (0.60, 1.04)
**Adult brining child to clinic**			
Mother/father	Reference	Reference	Reference
Grandparent	1.10 (0.93, 1.31)	**1.49 (1.25, 1.77)**	**1.45 (1.11, 1.79)**
Sibling	0.94 (0.60, 1.47)	1.27 (0.81, 1.98)	1.03 (0.42, 1.64)
Other family member	1.11 (0.66, 1.86)	1.42 (0.90, 2.25)	1.63 (0.61, 2.65)
Other adult (neighbor, orphanage caretaker)	0.99 (0.61, 1.58)	1.37 (0.83, 2.25)	1.90 (0.80, 3.01)
**Maternal antiretroviral treatment during pregnancy**	**0.81 (0.75, 0.87)**	**0.75 (0.69, 0.81)**	**0.84 (0.76, 0.91)**
**Child’s HIV status**			
HIV-exposed but uninfected	Reference	Reference	Reference
HIV-infected	**1.42 (1.25, 1.61)**	**2.15 (1.88, 2.47)**	**2.30 (1.96, 2.63)**
**Feeding solid foods at 3 months of age**	**0.84 (0.77, 0.93)**	**0.69 (0.61, 0.77)**	**0.84 (0.74, 0.94)**

Only variables reported were included within the adjusted odds ratio analysis.; Stunted- height-for-age Z-score ≤ -2; Underweight- weight-for-age Z-score ≤ -2; Wasted- weight-for-height Z-score ≤ -2; OR- odds ratio; 95%CI- 95% confidence interval

## Discussion

We found that, while the mean estimated z-scores for growth were generally above the cut-off for malnutrition, there were still a large proportion of children coming in each year who were malnourished. Some of the factors associated with malnourished status in children included living in urban settings, having a grandparent bring the child to clinic, and being HIV+. Females, those with *in-utero* ART exposure, and those who started mixed feeding by 3 months were less likely to be malnourished.

Malnutrition in the context of HIV is likely to have a ripple effect in increasing morbidities and mortalities in young children thus decreasing the gains made in reduction of under-five mortality globally over the last decade. Our results support prior findings of worse nutritional status in HIV+ children compared to HEU. In this cohort, HIV+ children were much more likely to be stunted, wasted, and underweight compared to HEU children. Even more concerning were the high proportion of both HIV+ and HEU children with moderate-to-severe stunting, underweight status, and wasting at each visit age. While our study did not include any HIV-unexposed children, national survey data from Kenya provides a comparative estimate of the general population’s nutritional status. According to the 2014 Kenya Demographic and Health Survey (KDHS), there was moderate-to-severe stunting in 35% of Kenyan children aged 12–17 months and 44% of Kenyan children aged 24–35 months [31]. Our study found rates of moderate-to-severe stunting 49% for HIV+ children at 12 months and 51% at 24 months. HEU children had similar rates of malnutrition compared to that reported in the KDHS survey. Similarly, there was a discrepancy between underweight and wasted status as reported within the KDHS compared to the HIV+ children within this clinical cohort [31]. Within the KDHS, the rates of malnutrition in the geographical regions where AMPATH operates did not differ substantially from the overall survey results, making it less likely that the difference found was primarily due to the regional rates of malnutrition. This leaves other factors, such as HIV infection, as being a potential contributor to worse nutritional status, as seen in other studies [8, 9, 18].

Our data have shown that HIV+ children are particularly vulnerable to malnutrition. Despite early initiation of ART, other studies from sub-Saharan Africa have also shown that HIV+ children have lower HFAZ compared to population norms [32, 33]. The etiology of this vulnerability is multifactorial, but certainly comorbidities such as increased prevalence of premature birth, diarrheal infection, pneumonia, tuberculosis infection, all contribute to the worse growth outcomes of HIV+ children [34–36]. While many of these comorbidities are also hold true for HEU children[13, 37], our study did not find differences in the proportion of children with malnutrition compared to national survey data. Growth data in other HEU populations within sub-Saharan Africa is mixed and it is unclear at this time whether there is a link between HIV exposure and malnutrition [18, 38, 39]. A meta-analysis is underway to determine across studies whether HEU children are also at higher risk for malnutrition [40]. Other social determinants of health, such as educational level and poverty, are also key variables that contribute to malnutrition, although they were not captured within this data set. We hypothesize these comorbidities play a role in malnutrition in our study population.

Our study also has the added advantage of tracking mean z-scores for children over time between the ages of 0–5 years. While there were decreases in mean z-scores around ages 1–2 years for both HIV+ and HEU children, the decrease in HIV+ typically occurred earlier. The etiology of this finding is unclear. However, we do know that failure to maintain adequate nutritional status during critical periods in brain development has lifelong effects on a child, and the first 1000 days of a child’s life are particularly important [41]. Thus, it is imperative that nutritional status is monitored from birth to ensure that interventions can be implemented prior to significant growth faltering.

The differences between the HIV+ and HEU children were clear early in analysis. Nearly all baseline characteristics differed significantly between the two groups. During comparisons of their rates of malnutrition and estimate mean scores, these differences remained. Due to the circumstances that allowed for the child to become HIV-infected, inherent differences exist between the groups including the age at enrollment and length of follow-up. For example, HIV+ children typically enrolled at an older age, likely due to the lack of maternal HIV diagnosis during pregnancy and hence, lack of ART exposure to prevent perinatal infection. Additionally, HEU children had better anthropometric outcomes compared to HIV+ children until approximately 18–24 months of age, at which time their estimated mean Z scores decreased. HIV+ and HEU children had similar rates of wasting above the age of 24 months and of stunting above the age of 36 months, with rates of wasted and stunted HEU children even exceeded that of HIV+ children in some cases. This suggests that HEU children remaining in care after 24 months of age may be inherently different than those lost-to-follow-up. We hypothesize that this is due to the confirmatory HIV testing which occurs at 18 months, and the large number of HEU children who stopped coming to clinic because, presumably, they were otherwise in generally good health. Those HEU children who remained to be seen within the clinic may have been sicker in general with poorer nutritional status, which is reflected in our results. These differences should be considered in any clinical setting that cares for both HIV+ and HEU children, to ensure appropriate resources are available at critical periods of need.

One area of concern that arose from this study was the discrepancy between the numbers of children identified as having failure-to-thrive by clinicians with the number of moderately-to-severely malnourished children. In settings with few providers trained in pediatrics, clinician assessments of growth, failure-to-thrive, and developmental delays might be very limited or absent. Even in well-resourced settings, failure-to-thrive in children is often overlooked [42]. However, in countries with minimal primary care services, healthcare systems that interact with children, either for acute conditions or specific disease processes, must educate their clinicians to identify and refer when issues are found to ensure optimal growth, health, and development of young children.

Other findings from this study provide new insights which warrant further exploration. In our study, females were less likely to be malnourished in stunting, underweight status, and wasting. The KDHS also indicated sex differences in regards to growth, with females faring better, and a meta-analysis found less stunting in girls in sub-Saharan Africa compared to boys [31, 43]. In other countries, such as India and Pakistan, studies have found the opposite to be true, with females being at greater risk for malnutrition [44, 45]. At this time, it is unclear what cultural or economic factors might account for these differences. However, it is important for countries to explore these differences to determine how to best improve the health of their children.

This study also found that children who started solid foods at 3 months were less likely to be malnourished. In this cohort, 1 in 6 HEU and 1 in 5 HIV+ children were started on solid foods at 3 months. The WHO and UNICEF recommend exclusive breastfeeding for the first 6 months of life to prevent undernutrition [46]. Some studies show exclusive breastfeeding for the first 6 months improves rates of stunting and underweight status [47]. However, other studies, such as a recent systematic review, did not find a difference in growth between feeding regimens, but it did show an improvement in the rate of iron deficiency anemia in breastfed infants when solid foods were introduced early [48]. For children born to HIV+ mothers, early mixed feeding is often discouraged due to the increased risk of HIV transmission and other infectious diseases [49, 50]. However, many HIV+ mothers start mixed feeding early, even in the first few weeks of an infant’s life [51]. While our results may suggest that early introduction to solid foods or mixed feedings benefits nutritional status, it is also possible that these children were introduced to solid foods earlier because their caregivers perceived that they were not gaining enough weight. More research is needed to clearly understand this issue before any definitive conclusions can be made.

Our study found that children were more likely to be malnourished when living in urban settings. This finding is in contrast with the KDHS data and other studies, which show that rural populations have increased rates of malnutrition compared to urban [31, 52]. However, these data often do not delineate individuals of lower socioeconomic status living in those urban settings, who often have much higher rates of malnutrition than other populations [53]. A study in Kenya showed that there is high prevalence of stunting (46%) in children living in urban poor settings [54]. We hypothesize that poverty in urban settings is a contributing factor to the outcomes of this study. Additionally, individuals living in rural areas may have the capabilities and resources to perform subsistence farming for some food items, whereas those living in urban settings would not. Healthcare systems must be aware of the disparities existing within urban populations to better identify children who are at risk for malnutrition and support them with nutritional services when warranted.

In this study, there were mixed results in terms of the nutritional status of children who were orphaned by either losing a single parent or both parents. Those children who had lost both parents tended to be less likely to have malnutrition than those who had both parents alive. One potential hypothesis for this finding may be that these children received additional resources through the healthcare system or government programs due to their vulnerable status. One limitation of this study is that we do not have data regarding nutritional referrals or receipt of food supplementation associated with each individual participant. Thus, we are only able to speculate on the results. While orphans are vulnerable to malnutrition, studies have found mixed results on this topic, and it appears the adverse effects of a parental loss may be mitigated if the appropriate resources and support are provided for the child [23, 55]. Child outcomes may also depend on who is ultimately responsible for the orphan’s care [56]. In our study, children brought to clinic by grandparents are more likely to be underweight and wasted, whereas no significant differences in nutritional status were noted when another family member brought the child to clinic. Further analyses would be necessary to delineate the potential heterogeneity among orphans in terms of their care provision and available resources. Other factors that may decrease the risk of malnutrition within our cohort should be explored.

With the nature of a retrospective chart review, there are limitations to the findings of this research. We relied on the data obtained at each clinic visit among 65 different clinics in western Kenya. While there are standard operating procedures for weighing and measuring the height of children during each visit, the methods and measurement instruments were not standardized throughout the data collection period as they would have been in a prospective trial. This may have introduced error into our data set. We attempted to mitigate that error by creating rules to remove data entries that fell out of the typical ranges of growth, but this would not have corrected for small variations in measurements. Another limitation to this study is that there was no HIV-unexposed group for comparison. During the time of data collection, only children exposed to HIV were seen within these clinics and data were subsequently entered into the EMR. The availability of the KDHS enables us to have a reference point of malnutrition rates in children of a similar age, living in the same regions as our AMPATH cohort, during the time range of our study’s data collection period. While the methods of collecting those data may differ slightly, this provides us with a representative population with whom we may compare our results. Additionally, due to the inherent differences between the HIV+ and HEU groups, these retrospective data reported may not be representative of the general characteristics of these populations as a whole. HIV+ children enrolled in clinic at an older age than HEU. As such, the characteristics of HIV+ children enrolling prior to 12 months of age may be different from those diagnosed later. Additionally, since a large number of HEU children no longer attended clinic after 18 months of age, the remaining cohort may not be representative of HEU between the ages of 24–60 months and may overestimate the proportion of children with malnutrition in those age groups. Finally, this study was also limited by the lack of differentiation among the HIV-indeterminate group, which was excluded from analysis. Because some members of that group may have been lost-to-follow-up prior to 18 months, their exclusion may lead to selection bias.

## Conclusions

This study demonstrated higher rates of stunting, underweight status, and wasting for children born to HIV+ mothers compared to external data from the general Kenyan population and identified factors associated with malnutrition in this healthcare system. Additionally, HIV+ infected children had higher rates of malnutrition at an earlier age compared to HEU children. To our knowledge, this is the first large retrospective study performed on malnutrition using an EMR system in a resource-limited setting. While the use of EMR data had its limitations, the knowledge obtained from the analysis of a large, clinical cohort of children is uniquely valuable and informs next steps needed to better understand and address malnutrition in this setting.

## Supporting information

S1 Table De-identified AMPATH data set used for analysis. (XLSX)
